# Editorial: Geometrical illusions: what they tell us about human vision in health and disease

**DOI:** 10.3389/fpsyg.2024.1439192

**Published:** 2024-06-25

**Authors:** Stephen Dopkins, Klaus Landwehr, Kyriaki Mikellidou

**Affiliations:** ^1^Department of Psychological and Brain Sciences, George Washington University, Washington, DC, United States; ^2^Department of Psychology, Johannes Gutenberg University Mainz, Mainz, Germany; ^3^Department of Psychology, University of Limassol, Nicosia, Cyprus

**Keywords:** geometrical illusions, visual perception, distance perception, perceptual errors, line length, separation, size perception, orientation

Oppel (1855) had defined geometrical illusions as judgmental errors about relative linear extents and the sizes of plane angles. Although this reference to Euclidean geometry may hold for many of the classical illusion figures, some of them may elicit impressions of depth (Ward et al., 1977), activating additional cognitive and neural processes. For the present Research Topic, we invited submissions based on a broad understanding of geometrical illusions. We were interested in new empirical findings as well as new approaches to theory using data from healthy participants as well as from clinical populations.

Kim et al. demonstrate several novel motion illusions that highlight the influence of relative size cues in the perception of depth. Their illusions are based on the Ames window illusion, for which the key stimulus is a window with greater horizontal than vertical extension, distorted such that the vertical frame is larger at one end than the other. The window is seen as it rotates in space around its vertical axis. Although the window rotates through 360 degrees, it is seen as rotating in one direction through ~180 degrees and then reversing and rotating in the other direction through ~180 degrees. Monocular cues dominate binocular cues. The illusion has been attributed to experience with a carpentered world. Kim et al. demonstrate several versions of the illusion that call this interpretation into question. The stimuli for the Kim et al. versions of the illusion lack angles or straight lines. Thus, a carpentered world interpretation struggles to accommodate the illusion occurring with these stimuli. Rather, the authors' results suggest that their version of the illusion reflects the influence of relative size depth cues in the perception of depth.

Hu et al. explore the illusory jitter occurring with Motion Induced Spatial Conflict (MISC), a phenomenon that arises when two rigid edges of a combined stimulus move at apparently different speeds. The authors show that the frequency of MISC jitter correlates across participants with the accrual rate of the Motion Induced Position Shift (MIPS), where the MIPS is the apparent shift that occurs in the static envelope of a grating when the grating moves within the envelope, and the accrual rate of this shift is the rate at which the shift increases in size with the duration of the grating stimulus. The authors suggest that the results reflect a common periodic process in the motion-based visual prediction that occurs with MISC and MIPS.

Lin et al. explore the connection between visual status, age, and the intensity of the Müller-Lyer illusion in congenitally visually impaired and visually healthy children aged 4–17 years. Additionally, Lin et al. conduct a comparative analysis of the developmental trends in the intensity of the illusion across three groups: visually impaired participants which utilized low vision aids (LVAs) (*N* = 53), visually impaired participants with no LVA experience (*N* = 72), and visually healthy participants (*N* = 133). Results show that children with congenital visual impairment experience the Müller-Lyer illusion more prominently compared to their visually healthy counterparts and this illusory experience becomes less prominent with increasing age across the three groups. Interestingly, some differences were also observed between the two visually impaired groups, with those lacking LVA use, experiencing greater illusory experience.

Wincza et al. seek to understand whether visual processing disturbances in Parkinson Disorder (PD) patients manifest through atypical experience of three visual illusions: Ebbinghaus, Ponzo, and Müller-Lyer. This study aimed to advance understanding of high-level perception in PD and the role of dopamine deficiency and basal ganglia pathophysiology documented in these patients in experiencing visual illusions. Their findings revealed no differences between PD patients (*N* = 28) and neurotypical controls (*N* = 28) matched on age, general cognitive abilities (memory, numeracy, attention, language), and mood. The lack of differences was especially evident in the Ebbinghaus and Müller-Lyer illusions that rely more strongly on context sensitivity rather than depth perception, suggesting that context integration (a key component of susceptibility to visual illusions) remains unaffected in the early to mid-stages of PD. A marginal indication of abnormalities in depth perception was indicated by reduced susceptibility to the Ponzo illusion, which is usually considered a classical illusion of depth.

Kirsch and Kunde revived the idea that, in addition to stimulus variables, variables pertaining to the observer might drive illusions. The authors describe several experiments they performed on a variety of geometrical illusion figures, aimed to show that attentional processes modulate effects. The hypothesis, in each case, was that attention may change receptive field sizes of cortical neurons, so that perceptual distortions would ensue. Kirsch and Kunde also consider specific stimulus features (e.g., spatial frequency) that might modulate effects, and they also discuss several geometrical illusions that do not appear amenable to their explanatory account.

In sum, the contributions of this Research Topic testify to the fact that geometrical visual illusions remain a useful, versatile tool to study human vision. Past research on visual illusions has helped understand the specialization of specific brain areas using neuroimaging techniques (Mikellidou et al., 2016) and has even challenged common fashion advice on how horizontally striped clothes can affect the perceived body size of a person (Thompson and Mikellidou, 2011). Although extensive investigations have been carried out to date aiming to unravel the mysteries behind visual illusions, many of them remain unexplained (Mikellidou and Thompson, 2013, 2014). Future research should strive to unravel in more detail the neural mechanisms responsible for the observed effects (Landwehr, 2022). In particular, possible interactions between the processing of different variables (e.g., size, plane interspaces, apparent distance, two/three-dimensional cues) need to be specified. Eventually, the exclusive focus on response bias should be supplemented by data on discriminative sensitivity which may accompany performance in parallel (Morgan et al., 1990; Dopkins and Galyer, 2020).

## Author contributions

SD: Writing – review & editing. KL: Writing – original draft. KM: Writing – review & editing.
